# Whole-Genome Sequencing (WGS) of Carbapenem-Resistant *K. pneumoniae* Isolated in Long-Term Care Facilities in the Northern Italian Region

**DOI:** 10.3390/microorganisms9091985

**Published:** 2021-09-17

**Authors:** Alessandra Piccirilli, Sabrina Cherubini, Anna Maria Azzini, Evelina Tacconelli, Giuliana Lo Cascio, Laura Maccacaro, Alda Bazaj, Laura Naso, Gianfranco Amicosante, LTCF-Veneto Working Group, Mariagrazia Perilli

**Affiliations:** 1 Department of Biotechnological and Applied Clinical Sciences, University of L’Aquila, 67100 L’Aquila, Italy; alessandra.piccirilli@univaq.it (A.P.); sabrina.cherubini@graduate.univaq.it (S.C.); gianfranco.amicosante@univaq.it (G.A.); 2 Infectious Disease Section, Department of Diagnostic and Public Health, University of Verona, 37134 Verona, Italy; annamaria.azzini@univr.it (A.M.A.); evelina.tacconelli@univr.it (E.T.); 3 Microbiology and Virology Unit, Department of Pathology and Diagnostic, Azienda Ospedaliera Universitaria Integrata di Verona, 37134 Verona, Italy; g.locascio@ausl.pc.it (G.L.C.); laura.maccacaro@aovr.veneto.it (L.M.); alda.bazaj@univr.it (A.B.); laura.naso@hotmail.it (L.N.); 4 Microbiology and Virology Unit, AUSL Piacenza, 29121 Piacenza, Italy

**Keywords:** *Klebsiella pneumoniae*, WGS, β-lactamases

## Abstract

*K. pneumoniae* (KPN) is one of the widest spread bacteria in which combined resistance to several antimicrobial groups is frequent. The most common β-lactamases found in *K. pneumoniae* are class A carbapenemases, both chromosomal-encoded (i.e., NMCA, IMI-1) and plasmid-encoded (i.e., GES-enzymes, IMI-2), VIM, IMP, NDM, OXA-48, and extended-spectrum β-lactamases (ESBLs) such as CTX-M enzymes. In the present study, a total of 68 carbapenem-resistant KPN were collected from twelve long-term care facilities (LTCFs) in the Northern Italian region. The whole-genome sequencing (WGS) of each KPN strain was determined using a MiSeq Illumina sequencing platform and analysed by a bacterial analysis pipeline (BAP) tool. The WGS analysis showed the prevalence of ST307, ST512, and ST37 as major lineages diffused among the twelve LTCFs. The other lineages found were: ST11, ST16, ST35, ST253, ST273, ST321, ST416, ST1519, ST2623, and ST3227. The *bla_KPC-_*_2_, *bla_KPC-_*_3_, *bla_KPC-_*_9_, *bla_SHV-_*_11_, *bla_SHV-_*_28_, *bla_CTX-M-_*_15_, *bla_OXA-_*_1_, *bla_OXA-_*_9_, *bla_OXA-_*_23_, *qnrS1*, *qnrB19*, *qnrB66*, *aac(6′)-Ib-cr*, and *fosA* were the resistance genes widespread in most LTCFs. In this study, we demonstrated the spreading of thirteen KPN lineages among the LTCFs. Additionally, KPC carbapenemases are the most widespread β-lactamase.

## 1. Introduction

β-lactams are the most widely prescribed antibiotics, used to treat a wide range of bacterial infections worldwide [1]. Since their introduction in clinical practice, resistance to β-lactams has progressively increased. Gram-negative bacteria resistance to β-lactams involves several factors such as (i) active efflux modification, (ii) decreased outer membrane permeability, (iii) mutations altering PBPs expression or function, and (iv) β-lactamases production, which remains the main mechanism of resistance employed by bacteria [2,3]. β-lactamases are usually divided into four classes, considering the Ambler classification: class A (TEM, SHV, ESBLs, and KPC); class B (MBL, NDM, VIM, and IMP); class C (Amp-C); and class D (OXA). Particularly widespread are the extended spectrum β-lactamases (ESBLs), against which the antibiotic class of carbapenems maintains their activity. However, carbapenemases, β-lactamases capable of hydrolysing even carbapenems, have emerged since 1982 in the *Serratia marcescens* clinical isolate (SME-1 carbapenemase) [4]. To date, serine- and metallo-carbapenemases are widely spread, especially among *Enterobacterales* [5]. The data from the European Antimicrobial Resistance Surveillance Network (EARS-Net) displayed that carbapenemase-producing *Enterobacterales* (CPE) are responsible for the majority of human infections [6]. Among *Enterobacterales*, *Klebsiella pneumoniae*, one of the most widely spread bacteria, is listed by the World Health Organization (WHO) as one of the worrisome pathogens in which combined resistance to several antimicrobials (β-lactams, quinolones, and aminoglycosides) is very frequent [7,8]. *K. pneumoniae* represents an important reservoir for class A (KPC, SHV, GES, IMI, and NMCA), class B (i.e., VIM, IMP, and NDM), class D carbapenemases (OXA-48), and extended-spectrum β-lactamases (ESBLs) such as CTX-M variants [5,9]. Carbapenem-resistant *K. pneumoniae* is largely disseminated around the world [10,11,12,13,14]. One reason for the rapid dissemination of *K. pneumoniae* is the presence of antibiotic resistance genes in mobile genetic elements and the Inc-groups, which belong to plasmids IncF, IncFII(K1), IncR, IncX, IncX3, IncI2, and ColE1 [15,16,17,18,19]. However, carbapenem-resistant *K. pneumoniae* strains are not only confined to healthcare settings, but also to the community and among residents of LTCFs [20,21,22,23,24]. In Italy, the carbapenem-resistant *Enterobacterales* from LTCFs have been reported since 2010 in different endemic areas [25,26,27,28]. The aim of the present study was to investigate the dissemination of antibiotic resistance genes in *K. pneumoniae* isolated from rectal swabs of residents in the LTCFs of the Veneto Region (Northern Italy). The molecular analysis of the WGS of these strains was performed by next-generation sequencing (NGS).

## 2. Materials and Methods

### 2.1. Setting

Between July 2018 and June 2019, we conducted a point-prevalence survey among the residents of 27 LTCFs in the Veneto Region, Northern Italy. Participation was on a voluntary basis. The study-specific data were collected on a single day for each LTCF involved; in the LTCFs with a high number of beds, data collection was spread over two or more consecutive days. However, all the beds in one ward were surveyed on the same day. The surveys were not conducted simultaneously in all 27 LTCFs, but at different periods for each facility in accordance with the availability of both the local staff to collaborate with researchers in the collection of clinical data and study-specific biological samples, and of the reference microbiology laboratory to accept samples and perform analysis.

Only subjects housed in the facility for at least 48 h were asked for consent to participate. A total of 118 variables, such as hospitalization and surgery during the previous year, antibiotics within the last three months, and the presence of medical devices (i.e., urinary catheter; peripheral vascular catheter; central venous catheter; nasogastric tube; and percutaneous endoscopic gastrostomy) were collected from each enrolled participant. Additionally, the enteric carriage of ESBL and carbapenemase-producing Gram-negative bacteria was assessed collecting a rectal swab from every enrolled resident.

### 2.2. Strains Selection

The rectal swabs were collected and inoculated onto ChromID ESBL agar (bioMerieux, Marcy l’Etoile, France) with an Ertapenem disk (10 μg) and on Mac Conkey agar with a Meropenem disk (10 μg). The plates were incubated at 35 ± 2 °C under aerobic conditions for 24 h. The isolates were identified at the species level using an automated Vitek2 System (bioMerieux, Marcy l’Etoile, France). Resistance to carbapenems were interpreted according to the EUCAST criteria and confirmed with an immunochromatographic lateral flow assay Carba5 (NG Biotech, Guipry, France). A Vitek2 system (version 9.02, bioMérieux, Marcy l’Etoile, France) was used to confirm carbapenem resistance and to perform antimicrobial susceptibilities for other substances.

### 2.3. DNA Extraction and Whole-Genome Sequencing (WGS)

Total nucleic acid was extracted using a MagMAX Microbiome Ultra Nucleic Acid Isolation kit (Applied Biosystems and ThermoFisher Scientific, Monza, Italy). The DNA concentrations were measured using a Qubit fluorometer (ThermoFisher Scientific) to determine DNA input. The genomic libraries were prepared using a Swift 2S Turbo DNA Library kit (Swift Biosciences, Ann Arbor, MI, USA) and the WGS was performed on an Illumina MiSeq platform using v3 reagent kits generating 2 × 300 bp paired-end reads (Illumina, San Diego, CA, USA).

### 2.4. Bioinformatic Analysis

Raw data from the paired-end sequencing were quality checked with the FastQC tool (v.0.11.6, BaseSpaceLabs, Illumina, San Diego, CA, USA) and assembled with Velvet (v.1.2.10, BaseSpaceLabs, Illumina, San Diego, CA, USA) [29]. Velvet is incorporated as an assembler in a multiple-tool workflow, the CGE Bacterial Analysis Pipeline (BAP) (BaseSpaceApps, Illumina, San Diego, CA, USA). The BAP application predicts the species of bacterial input genomes using a *k*-mer-based approach [30]. These acquired antimicrobial resistance genes were identified using a BLAST-based approach, where the nucleotide sequence of the input genome was compared to the genes in the ResFinder database [31]. Multilocus sequence typing (MLST) was performed also using a BLAST-based approach [32]. BLAST was used to search for plasmid replicons using the PlasmidFinder database [33]. Identified plasmids of the IncF, IncH1, IncH2, IncI1, IncN, or IncA/C type were subtyped by plasmid MLST [33]. KmerFinder, ResFinder, and PlasmidFinder databases were synchronized with databases from the Center for Genomic Epidemiology (http://www.genomicepidemiology.org/) on 2 June 2017. MLST and pMLST data were downloaded from pubmlst.org on 17 May 2017.

## 3. Results

In total, 1933 rectal swabs in 2890 residents were performed. Overall, of 159 *K. pneumoniae* (KPN), isolated on a selective medium, only 68 of them were selected for this study for their carbapenem resistance profile. As shown in Table 1, all strains were resistant to at least two different class of antibiotics. Indeed, they exhibited a high resistance profile to β-lactams/β-lactamase inhibitors (amoxicillin-clavulanic acid and piperacillin-tazobactam), oxyiminocephalosporins (cefotaxime and ceftazidime), carbapenems (meropenem and ertapenem), and ciprofloxacin. Of 68 KPN, 36 (52.9%) were resistant to trimethoprim-sulfamethoxazole, respectively. All strains, analysed by Vitek2 system, were susceptible to colistin.

For legal aspects, we used only the acronym of the twelve LTCFs.

### 3.1. WGS of K. pneumoniae

Whole-genome sequencing was carried out on 68 KPN isolates, and bioinformatic analysis was performed with a BAP tool that gave information about genome size, MLST, plasmid replicons, pMLST, and antimicrobial resistance genes. The genome size of the 68 KPN ranged from 5.1 to 5.74 Mb.

### 3.2. K. pneumoniae MLST

**Figure 1:**
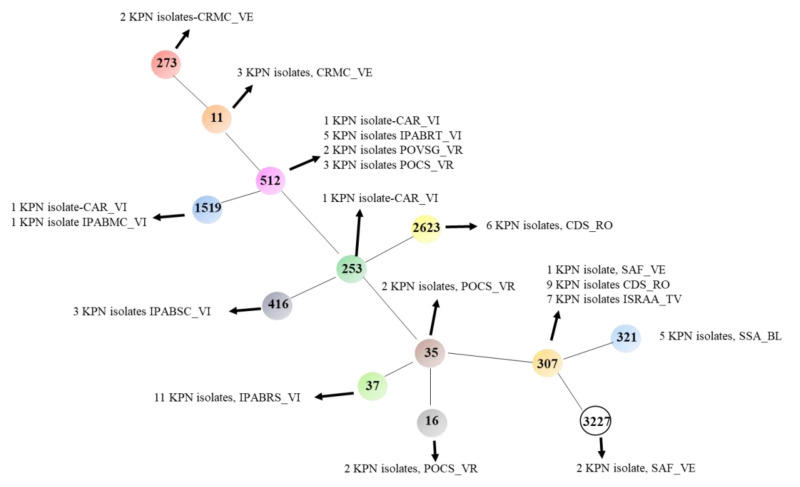
Phylogenetic tree of *K. pneumoniae* strains selected for MLST. The minimum spanning tree was obtained using PHYLOViZ online software (http://www.phyloviz.net/). For legal aspects, we used only the acronym of the twelve LTCFs.

The MLST analysis showed the presence of thirteen KPN lineages: ST11, ST16, ST35, ST37, ST253, ST273, ST307, ST321, ST416, ST512, ST1519, ST2623, and ST3227. The most widespread STs were ST307 (17 isolates), ST512 (11 isolates), and ST37 (11 isolates). The ST307, ST512, and ST37 were identified in four, three, and one LTCFs, respectively. The remaining STs were endemic of only one LTCF (Figure 1, Table 2).

### 3.3. Plasmids Replicons and pMLST

**Figure 2:**
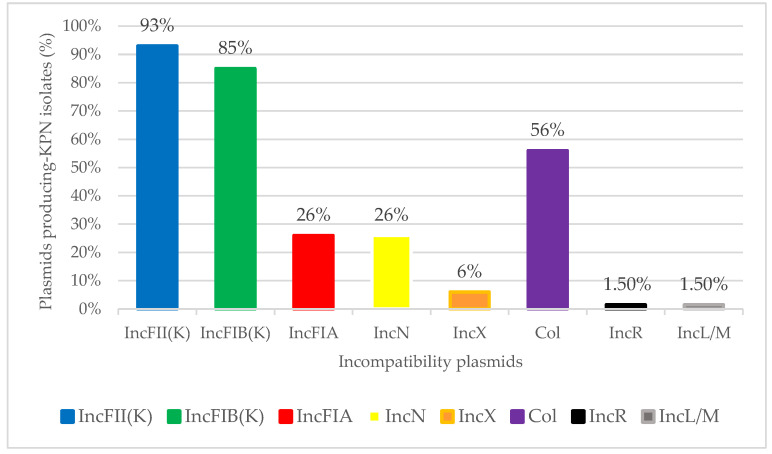
Distribution of incompatibility plasmids found in 68 *K. pneumoniae* isolated from residents of twelve LTCFs (Northern Italy).

Incompatibility plasmids IncFII(K), IncFIB(K), IncFIA(HI1), IncN, IncF, IncFIB (pQil), IncL/M, IncX3, IncX4, Col (MG828), ColpVC, and ColRNAI were detected in all KPN analysed (Figure 2). Overall, IncF was the predominant plasmid found in 100% of the KPN, followed by Col (38 out of 68 KPN), IncN (18 out of 68 KPN), IncX (4 out of 68 KPN), IncR (1 out of 68 KPN), and IncL/M (1 out of 68 KPN) type plasmids. Among IncF plasmids, the predominant was IncFII(K) (found in 63 out of 68 KPN), followed by IncFIB(K) (found in 52 out of 68 KPN), and IncFIA (found in 18 out of 68 KPN). Regarding pMLST, the major plasmid lineage was fiik7 (25 KPN isolates), followed by fiik1 (11 isolates), fia19 (9 isolates), fia10 (8 isolates), fiik5 (4 isolates), fiik2 (2 isolates), fiik4 (1 isolate), fiik9 (1 isolate), and fia18 (1 isolate). In most KPNs, the simultaneous presence of more than one plasmid was found.

### 3.4. β-Lactam Resistance Genes

The β-lactam resistance genes were found in all KPNs with a moderate variability among the LTCFs (Table 2). The prototype genes *bla_TEM-_*_1*A*_/*bla_TEM-_*_1*B*_ were found in 65% of KPN isolates, whereas the *bla_SHV_* variants (*bla_SHV-_*_1_, *bla_SHV-_*_11_, *bla_SHV-_*_14_, *bla_SHV-_*_28_, *bla_SHV-_*_33_, *bla_SHV-_*_36_, and *bla_SHV-_*_99_) were found in 76% of KPN strains (52 out of 68) isolated in the twelve LTCFs with a prevalence of *bla_SHV-_*_11_ (31% of isolates), followed by *bla_SHV-_*_28_ (25% of isolates) (Table 3). *bla_KPC-_*_2_ and *bla_KPC-_*_3_ were found in 47 out of 68 isolates (69%) collected in 9 to 12 LTCFs. *bla_KPC-_*_2_ was retrieved in 17 isolates belonging to ST37 and ST307, whereas *bla_KPC-_*_3_ was found in 30 KPNs belonging to seven different ST lineages (Table 3). *bla_KPC-_*_9_ was found in two KPN isolates (ST512 and ST3227 lineages) collected from two different LTCFs. The OXA variants were the predominant β-lactamase found in 93% of KPNs (63 out of 68 isolates). In detail, OXA-1, OXA-9, and OXA-1/OXA-9 associations were found in 20, 13, and 20 KPN isolates, respectively. *bla_OXA-_*_23_ was identified in two ST512 isolated from two residents of the same LTCF. The *bla_LEN-_*_7_ and *bla_LEN-_*_12_ genes were found in 11 KPN isolates (ST512 and ST3227 lineages) in four of the LTCFs. *bla_CTX-M-_*_15_ was found in 35 out of 68 KPNs, belonging to eight different lineages (Table 3). The metallo-β-lactamases, VIM-1 and NDM-1, were detected in nine and three KPN isolates, respectively. The three NDM-1-producing KPNs were identified only in one LTCF. The *bla_OXA-_*_23_ gene was found in two ST512 isolates in combination with *bla_CTX-M-_*_15_, *bla_OXA-_*_1_, and *bla_LEN-_*_12_.

### 3.5. Fluoroquinolone Resistance Genes

Plasmid-mediated resistance to fluoroquinolones was identified in all KPNs. Different plasmid-mediated mechanisms were implicated in quinolone resistance: (i) *aac(6′)Ib-cr* in 100% of KPNs, (ii) *qnr* elements in 58% of KPNs, and (iii) *oqxAB* multidrug efflux pump was detected in 53 KPN isolates disseminated in ten LTCFs. Among the *qnr* elements, the major *qnr* found were *qnrB66* (in 26 out of 68 KPNs), followed by *qnrS1* (9 out of 68 KPNs), *qnrB6* (one isolate), and *qnrB19* (one isolate).

### 3.6. Other Antimicrobial Resistance Genes

Several molecular mechanisms of sulfamethoxazole/trimethoprim resistance have been described in the literature; however, the most common mechanism is the acquisition of dihydrofolate reductase *dfr*. Indeed, 54 out of 68 strains that were analyzed showed the presence of *dfrA12*, *dfrA14,* and *dfrA30*. In detail, 4 KPNs showed *dfrA12*, 39 KPNs showed *dfrA14,* and 11 KPNs presented both *dfrA14* and *dfrA30*. Resistance to aminoglycosides was mediated by *aadA1*, *aadA2*, *aph(3′)-XV*, *aacA4*, *aph(3′)-Ia*, and *aph(3′)-IIa,* and by the bi-functional gene *aac(6′)Ib-cr*, which confers resistance to both fluoroquinolones and aminoglycosides. Other antibiotic resistance genes detected in KPN were the following: *mphA* (macrolide resistance) detected in 8 KPNs; *sul1* and *sul2* (sulfonamide resistance) detected in 22 and 20 KPNs, respectively; *catA1*, *catB2*, and *catB4* (chloramphenicol resistance) detected in 50 KPNs; *strA* and *strB* (streptomycin resistance) detected in 23 KPNs; *tet*(A), *tet*(B), and *tet*(D) (tetracycline resistance) detected in 13 KPNs; and *fosA* gene (fosfomycin resistance) detected in 61 KPNs.

## 4. Discussion

Carbapenem-resistant *K. pneumoniae* is designated by the Centres for Disease Control and Prevention (CDC) as one of the microorganisms that poses an urgent threat to public health worldwide. *K. pneumoniae* can spread rapidly in healthcare settings, and it is responsible for numerous human infections such as urinary, respiratory, and bloodstream infections [34,35]. In Italy, an epidemic spread of *K. pneumoniae* ST258, as a major contributor of carbapenem-resistant *Enterobacterales*, has been observed since 2010 [36,37]. In parallel, even though most infections still occur in nosocomial settings, *K. pneumoniae* emerged as a cause of severe community-acquired infections. The present study investigated the genome of carbapenem-resistant KPN in twelve LTCFs in a Northern Italy region. Among the 68 carbapenem-resistant KPNs, the most widespread clones were represented by ST307 and ST512. In ISRAA_TV LTCF, the ST307 was the unique lineage found, and it harbors the same plasmids but different resistance genes. *K. pneumoniae* ST307 has been reported from many countries, and it has been responsible for several global nosocomial [38,39] and long-term care center outbreaks [40,41]. Whole-genome sequencing performed by Wyres et al. on 95 *K. pneumoniae* ST307 revealed the presence of FIB-like plasmids harbouring the *bla_CTX-M-_*_15_ gene adjacent to the IS*ECp1* element such as the other ST307 isolated in different geographical areas [38,42]. The ST307 harbouring *bla_CTX-M-_*_15_ in association with *aac(6′)-Ib-cr* and *qnrB6* genes, as well as in our strains, was also described in an Italian regional survey (Sicily, Southern Italy) [27].

In the present study, *K. pneumoniae* ST512, found in four LTCFs, harboured KPC-3 and KPC-9, CTX-M-15, SHV-11, OXA-9, OXA-23, and LEN-12 β-lactamases. The carbapenem-resistant *K. pneumoniae* ST512 has been considered the predominant lineage in isolates, causing severe bloodstream infections in a Northern Italian hospital [43]. For a long time, Italy has been an endemic country for *K. pneumoniae* ST258/ST512 lineages [36,37,43,44,45], but recently, other STs emerged [46]. The *K. pneumoniae* ST37 was the third most spread lineage (11 isolates) but it was detected only in one LTCF (IPABRS_VI). A determination of the correlation between this evidence and the presence of specific risk factors in the reference population is underway. The ST37 has been described in several papers as an ertapenem-resistant *K. pneumoniae* with a modified outer membrane permeability [47,48].

A wide variety of plasmids were found in each whole KPN genome. The IncF plasmids were predominant in the KPN analysed in this study. This plasmid family is widely diffused in clinically relevant *Enterobacterales,* especially IncFII(K), which is considered a virulent plasmid because of its ability to co-exist with other plasmids in a single cell [49,50]. Most of these plasmids are conjugative and this facilitates the dissemination of resistance genes among different strains and species. This is the case of IncFIA(pBK30683 plasmid found in ST512 (POCS_VR LTCF), co-harbouring *bla_OXA-_*_23_, *bla_OXA-_*_1_, *bla_CTX-M-_*_15_, and *bla_LEN-_*_12_. The OXA-23 class D carbapenemase is normally produced by *A. baumannii,* and it is very rare in *K. pneumoniae* [51,52]. However, pBK30683 is a plasmid of 139,941 bp that seems to originate from the cointegration of pBK30661 (belonging to IncFIA family plasmids) with a 68 Kb genetic element, harbouring a complete set of genes for plasmid replication, stability, and conjugation [53]. This could explain the “jump” of *bla_OXA-_*_23_ from one species to another.

Class A, B, and D β-lactamases were identified in all the KPN strains. The major carbapenemases were the KPC variants (KPC-2, KPC-3, and KPC-9), VIM-1, NDM-1 metallo-β-lactamases, and OXA-23. KPC-9 is a KPC-3 variant with a V239A substitution. CTX-M-15 is the most common ESBL in *K. pneumoniae*. In our strains, CTX-M-15 was identified in eight different ST lineages (11, 16, 37, 273, 307, 512, 2623, and 3227). The *bla_CTX-M-_*_15_ gene is often flanked by a sequence insertion (IS) such as IS*Ecp1*, which facilitates its mobility. The β-lactamases LEN and OKP-B are chromosomally encoded and are frequently found in *K. pneumoniae* as well as the oxacillinases (OXA-1 and OXA-9).

## 5. Conclusions

This study found that the LTCFs represent an important incubator for class A, B, and D carbapenemases, ESBLs, and other antibiotic resistance genes, reflecting the local hospital trends. In many cases, the total dependence of residents on nurse care for their daily living activities exposes them to both the selection and horizontal transmission of antibiotic-resistant organisms. It is important for LTCFs to develop effective control measures to prevent outbreaks of antibiotic-resistant strains.

## Figures and Tables

**Table 1:** Antimicrobial susceptibility of 68 *K. pneumoniae* isolated from rectal swabs.

LTCFs	No. Isolates	ST	Resistance Profile
CDS_RO	15	307, 2623	AMX, TZP, CTX, CAZ, ERT, MEM, CIP
POCS_VR	2	16	AMX, TZP, CTX, CAZ, ERT, MEM, CIP, SXT
	2	35	AMX, TZP, CTX, CAZ, CIP, MEM, SXT
	3	512	AMX, TZP, CTX, CAZ, ERT, MEM, CIP
POVSG_VR	2	512	AMX, TZP, CTX, CAZ, ERT, MEM, CIP
IPABMC_VI	2	253, 1519	AMX, TZP, CTX, CAZ, ERT, MEM, CIP, SXT
CAR_VI	2	512, 1519	AMX, TZP, CTX, CAZ, ERT, MEM, CIP, SXT
IPABRS_VI	11	37	AMX, TZP, CTX, CAZ, ERT, MEM, CIP, SXT
IPABRT_VI	5	512	AMX, TZP, CTX, CAZ, ERT, MEM, CIP, SXT
IPABSC_VI	3	416	AMX, TZP, CTX, CAZ, MEM, CIP, SXT
SSA_BL	5	321	AMX, TZP, CTX, CAZ, MEM, CIP, SXT
ISRAA_TV	7	307	AMX, TZP, CTX, CAZ, ERT, MEM, CIP, SXT
CRMC_VE	5	11, 273	AMX, TZP, CTX, CAZ, ERT, MEM, CIP, SXT
SAF_VE	4	307	AMX, TZP, CTX, CAZ, ERT, MEM, CIP, SXT

AMX, Amoxicillin-clavulanic acid; TZP, piperacillin-tazobactam; CTX, cefotaxime; CAZ, ceftazidime; ERT, ertapenem; MEM, meropenem; CIP, ciprofloxacin; SXT, trimethoprim-sulfamethoxazole. ST, sub type.

**Table 2:** Genome analysis of *K. pneumoniae* isolated from residents of twelve LTCFs (Northern Italy).

LTCFs	No. Isolates	Genome Size (bp)	MLST	Plasmid Replicons/pMLST	β-lactams Resistance Genes	Other Antibiotics Resistance Genes
SSA_BL	5	5,736,085	ST321	IncFII(K), IncFIB(K), IncFIA(HI1), IncN/fii_k1 fia_19	*bla_VIM-_*_1_, *bla_SHV-_*_99_	*aadA1*, *aph(3′)-XV*, *aacA4*, *fosA*, *mph(A)*, *aac(6′)Ib-cr,oqxA*, *oqxB*, *qnrS1*, *sul1* *dfrA14*, *catB2*
ISRAA_TV	2	5,497,503	ST307	IncFIB(K), IncFII(K)/fii_k7	*bla_SHV-_*_28_, *bla_KPC-_*_2_, *bla_OXA-_*_9_, *bla_TEM-_*_1*A*_, *bla_CTX-M-_*_15_	*fosA*, *dfrA14*, *qnrB66*
	2	5,164,710	ST307	IncFIB(K), IncFII(K)/fii_k7	*bla_KPC-_*_2_, *bla_TEM-_*_1*A*_, *bla_OXA-_*_9_, *bla_CTX-M-_*_15_, *bla_SHV-_*_28_, *bla_OXA-_*_1_	*aac(6′)-Ib-cr*, *fosA*, *dfrA14*, *qnrB66*, *catB4*
	2	5,578,258	ST307	IncFIB(K), IncFII(K)/fii_k7	*bla_KPC-_*_2_, *bla_TEM-_*_1*A*_, *bla_OXA-_*_9_, *bla_CTX-M-_*_15_, *bla_SHV-_*_28_, *bla_OXA-_*_1_	*aac(6′)-Ib-cr*, *fosA*, *dfrA14*, *qnrB66*, *catB4*, *oqxA*, *oqxB*
	1	5,505,674	ST307	IncFIB(K), IncFII(K)/fii_k7	*bla_KPC-_*_2_, *bla_TEM-_*_1*A*_, *bla_OXA-_*_9_, *bla_CTX-M-_*_15_, *bla_SHV-_*_28_, *bla_OXA-_*_1_	*aac(6′)-Ib-cr*, *fosA*, *dfrA14*, *catB4*, *oqxA*, *oqxB*
SAF_VE	1	5,545,376	ST307	IncFIB(K), IncFII(K)/fii_k7	*bla_KPC-_*_2_, *bla_TEM-_*_1*A*_, *bla_OXA-_*_9_, *bla_CTX-M-_*_15_, *bla_SHV-_*_28_, *bla_OXA-_*_1_	*aac(6′)-Ib-cr*, *fosA*, *dfrA14*, *qnrB66*, *catB4*, *oqxA*, *oqxB*
	1	5,330,546	ST3227	IncFIB(pQil), IncFIB(K)/fii_k7	*bla_KPC-_*_9_, *bla_TEM-_*_1*B*_, *bla_OXA-_*_9_, *bla_LEN-_*_12_, *bla_CTX-M-_*_15_, *bla_OXA-_*_1_	*aph(3′)-IIa*, *aac(6′)-Ib-cr*, *fosA*, *strA*, *strB*, *sul2*, *dfrA14*, *qnrB66*, *catB4*
	1	5,545,568	ST3227	IncFIB(pQil), IncFII(K), IncFIB(K)/fii_k9	*bla_OXA-_*_9_, *bla_TEM-_*_1*A*_, *bla_KPC-_*_3_, *bla_LEN-_*_12_, *bla_LEN-_*_7_, *bla_OXA-_*_1_	*aac(6′)-Ib-cr*, *aph(3′)-IIa*, *fosA*, *dfrA14*, *oqxB*, *qnrB66*, *catB4*
	1	5,540,988	ST307	IncFII(K), IncFIB(K), IncR, IncL/M (pOXA-48)/fii_k5	*bla_TEM-_*_1*A*_, *bla_SHV-_*_11_	*aph(3′)-Ia*, *fosA*, *qnrB19*, *oqxB*
CRMC_VE	3	5,531,831	ST11	IncFII(pKPX1), ColpVC, Col(MG828)/fii_k7	*bla_SHV-_*_11_, *bla_CTX-M-_*_15_, *bla_NDM-_*_1_	*aac(6′)-Ib-cr*, *fosA*, *oqxB*, *oqxA*
	2	5,387,384	ST273	IncFII(K), IncFIB(K), Col(MG828)/fii_k7	*bla_SHV-_*_11_, *bla_TEM-_*_1*B*_, *bla_OXA-_*_1_, *bla_CTX-M-_*_15_	*aac(3′)-IIa*, *aac(6′)-Ib-cr*, *strA*, *strB*, *fosA*, *sul2*, *dfrA14*, *oqxA*, *oqxB*, *catB4*
CDS_RO	3	5,424,562	ST2623	IncFII(K), IncFIB(K), IncFIA(HI1), ColRNAI/fii_k1 fia_10	*bla_CTX-M-_*_15_, *bla_SHV-_*_1_, *bla_TEM-_*_1*B*_, *bla_OXA-_*_1_	*aac(6′)-Ib-cr*, *strA*, *strB*, *sul2* *oqxA*, *oqxB*, *qnrB66*, *tet(D)*, *catB4*
	3	5,666,417	ST 2623	IncFIB(pQil), IncFII(K), IncFIB(K), IncFIA(HI1), ColRNAI/fii_k1 fia_10	*bla_CTX-M-_*_15_, *bla_OXA-_*_9_, *bla_TEM-_*_1*B*_, *bla_OXA-_*_1_, *bla_KPC-_*_3_	*aac(6′)-Ib-cr*, *fosA*, *strA*, *strB*, *sul2*, *oqxA*, *oqxB*, *qnrB66*, *tet(D)*, *catB4*
	2	5,607,404	ST307	IncFIB(pQil), IncFII(K), IncFIA(HI1)/fii_k2 fia_19	*bla_TEM-_*_1*A*_, *bla_OXA-_*_9_, *bla_SHV-_*_28_, *bla_CTX-M-_*_15_, *bla_OXA-_*_1_, *bla_KPC-_*_3_	*aac(6′)-Ib-cr*, *strA*, *strB*, *fosA*, *sul2*, *oqxA*, *oqxB*, *qnrB66*, *catB4*
	37	5,496,232	ST307	IncFIB(pQil), IncFII(K)/fii_k2	*bla_CTX-M-_*_15_, *bla_TEM-_*_1*A*_, *bla_OXA-_*_9_, *bla_SHV-_*_28_, *bla_OXA-_*_1_, *bla_KPC-_*_3_	*aac(6′)-Ib-cr*, *fosA*, *sul2*, *oqxA*, *oqxB*, *qnrB66*, *catB4*
	2	5,566,824	ST307	IncFIB(pQil), IncFII(K), IncFIA(HI1)/fii_k2 fia_19	*bla_CTX-M-_*_15_, *bla_TEM-_*_1*A*_, *bla_OXA-_*_9_, *bla_SHV-_*_28_, *bla_OXA-_*_1_, *bla_KPC-_*_3_	*aac(6′)-Ib-cr*, *strA*, *strB*, *fosA*, *sul2*, *oqxA*, *oqxB* *qnrB66*, *catB4*
	2	5,634,072	ST307	IncFIB(pQil), IncFII(K), IncFIB(K)/fii_k2	*bla_CTX-M-_*_15_, *bla_TEM-_*_1*A*_, *bla_OXA-_*_9_, *bla_SHV-_*_28_, *bla_OXA-_*_1_, *bla_KPC-_*_3_	*aac(6′)-Ib-cr*, *aac(3)-IIa*, *strA*, *strB*, *fosA*, *sul2*, *dfrA14*, *oqxA*, *oqxB*, *qnrB66*, *catB4*
CAR_VI	1	5,649,046	ST512	IncX3, IncFIB(K), IncFIB(pQil) IncFII(K)/fii_k2	*bla_KPC-_*_3_, *bla_TEM-_*_1*A*_, *bla_OXA-_*_9_, *bla_SHV-_*_11_	*aadA2*, *aph(3′)-Ia*, *fosA*, *mph(A)*, *sul1*, *dfrA12*, *oqxA*, *oqxB*, *catA1*
	1	5,487,176	ST1519	ColRNAI, IncFIB(pQil), IncFII(K)/fii_k2	*bla_KPC-_*_3_, *bla_OXA-_*_9_, *bla_SHV-_*_11_, *bla_TEM-_*_1*A*_	*aadA2*, *aac(6′)-Ib-cr*, *fosA*, *sul1*, *oqxA*, *oqxB*
IPABMC_VI	1	5,311,196	ST1519	ColRNAI, IncFIB(pQil), IncFII(K), IncFIB(K)/fii_k2	*bla_SHV-_*_11_, *bla_KPC-_*_3_	*aac(6′)-Ib-cr*, *aadA2*, *fosA*, *mph(A)*, *sul1*, *dfrA12*, *oqxB*, *catA1*
	1	5,651,358	ST253	ColRNAI, IncFIB(pQil), IncFII(K), IncFIB(K), IncN/ fii_k5/ST7	*bla_VIM-_*_1_, *bla_SHV-_*_36_	*aac(6′)-Ib-cr*, *aadA1*, *aacA4*, *fosA*, *sul1*, *dfrA14*, *qnrS1*, *catB2*
IPABRS_VI	2	5,167,604	ST37	ColRNAI, IncFII(K), IncFIB(K)/fii_k7	*bla_CTX-M-_*_15_, *bla_SHV-_*_11_, *bla_OXA-_*_1_, *bla_TEM-_*_1*B*_	*aac(6′)-Ib-cr*, *strA*, *strB*, *fosA*, *sul2*, *dfrA14*, *oqxA*, *oqxB*, *catB4*
	4	5,451,323	ST37	ColRNAI, IncFIB(pQil), IncFII(K), IncFIB(K), IncN/ fii_k7	*bla_SHV-_*_11_, *bla_OXA-_*_1_, *bla_KPC-_*_2_	*aac(6′)-Ib-cr*, *fosA*, *dfrA14*, *dfrA30*, *oqxB*, *oqxA*, *catB4*
	3	5,424,901	ST37	ColRNAI, IncFIB(pQil), IncFII(K), IncFIB(K), IncN/ IncF: fii_k7 IncN: unknown	*bla_SHV-_*_11_, *bla_OXA-_*_1_, *bla_KPC-_*_2_	*aac(6′)-Ib-cr*, *fosA*, *dfrA30*, *dfrA14*, *oqxA*, *oqxB*, *catB4*
	2	5,444,858	ST37	ColRNAI, IncFIB(pQil), IncFII(K), IncFIB(K), IncN/ IncF: fii_k7	*bla_SHV-_*_11_, *bla_OXA-_*_1_, *bla_KPC-_*_2_	*aac(6′)-Ib-cr*, *fosA*, *dfrA30*, *dfrA14*, *oqxA*, *oqxB*, *catB4*
IPABRT_VI	5	5,406,932	ST512	ColRNAI, IncFIB(pQil), IncFII(K), IncFIB(K)/fii_k2	*bla_LEN-_*_12_, *bla_TEM-_*_1*A*_, *bla_KPC-_*_3_, *bla_OXA-_*_9_	*aac(6′)-Ib-cr*, *aadA1*, *fosA*, *sul1*, *dfrA1*, *oqxB*, *tet(D)*
IPABSC_VI	1	5,634,812	ST416	IncFII(K), IncFIA(HI1), ColRNAI, IncN, IncFIB(K)/ IncF: fii_k4, fia_18 IncN: ST7	*bla_VIM-_*_1_, *bla_SHV-_*_14_	*aadA1*, *aph(3′)-XV*, *aacA4*, *fosA*, *sul1*, *dfrA14*, *oqxA*, *oqxB*, *qnrS1*, *catB2*
	2	5,477,368	ST416	IncFII(K), IncFIB(Mar), ColRNAI, IncN, IncFIB(K)/ IncF: fii_k5 IncN: ST7	*bla_VIM-_*_1_, *bla_OKP-B-_*_3_	*aadA1*, *aph(3′)-XV*, *aacA4*, *aac(6′)-Ib-cr*, *fosA*, *sul1*, *qnrS1*, *catB2*, *oqxB*, *oqxA*
POVSG_VR	1	5,346,528	ST512	ColRNAI, IncFIB(K)/fii_k2	*bla_LEN-_*_7_, *bla_OXA-_*_9_, *bla_TEM-_*_1*A*_	*aph(3′)-IIa*, *aadA2*, *aac(6′)-Ib-cr*, *fosA*, *sul1*
	1	5,101,877	ST512	ColRNAI, IncFIB(pQil), IncFII(K), IncFIB(K)/fii_k2	*bla_KPC-_*_9_, *bla_OXA-_*_9_, *bla_TEM-_*_1*A*_	*aph(3′)-IIa*, *aadA2*, *aac(6′)-Ib-cr*, *fosA*, *sul1*
POCS_VR	1	5,548,552	ST512	IncFIB(pQil), IncFII(K), IncFIB(K), ColRNAI, IncX3/ fii_k2	*bla_OXA-_*_9_, *bla_SHV-_*_11_, *bla_TEM-_*_1*A*_, *bla_KPC-_*_3_	*aadA2*, *aac(6′)-Ib-cr*, *mph(A)*, *sul1*, *dfrA12*, *oqxB*, *oqxA*, *catA1*
	2	5,589,508	ST512	IncFII(K), IncFIA(HI1), ColRNAI, IncFIB(K), Col(MG828), FIA(pBK30683)/fii_k12 fia_10	*bla_OXA-_*_23_, *bla_LEN-_*_12_, *bla_CTX-M-_*_15_, *bla_OXA-_*_1_	*aac(3)-IIa*, *aac(6′)-Ib-cr*, *aadA1*, *fosA*, *sul1*, *dfrA1*, *oqxB*, *qnrB6*, *tet(D)*, *tet(B)*, *catB4*, *catA1*
	2	5,604,773	ST16	IncFIB(pQil), IncFII(K), IncFIB(K), IncX4/fii_k2	*bla_KPC-_*_3_, *bla_CTX-M-_*_15_, *bla_TEM-_*_1*B*_, *bla_SHV-_*_1_, *bla_OXA-_*_1_	*strA*, *aac(6′)-Ib-cr*, *strB*, *fosA*, *sul2*, *dfrA14*, *oqxB*, *oqxA*, *qnrB66*, *tet(A)*, *catB4*
	2	5,589,102	ST35	IncFIB(pQil), IncFII(K), IncFIB(K)/fii_k2	*bla_TEM-_*_1*A*_, *bla_OXA-_*_9_, *bla_KPC-_*_3_, *bla_SHV-_*_33_	*fosA*, *oqxB*, *oqxA*

For legal aspects, we only used the acronym of the twelve LTCFs.

**Table 3:** Distribution of β-lactamases among *K. pneumoniae* isolated from residents of 12 LTCFs (Northern Italy).

β-lactamases	Classes	Isolates No. (%)	ST
TEM-1	A	44 (65)	16, 35, 37, 273, 307, 512, 1519, 2623, 3227
KPC-2	A	17 (25)	37, 307
KPC-3	A	30 (44)	16, 35, 307, 512, 1519, 2623, 3227
KPC-9	A	2 (3)	512, 3227
SHV-1	A	5 (7)	16, 2623
SHV-11	A	21 (31)	11, 37, 273, 307, 512, 1519
SHV-14	A	1 (1.5)	416
SHV-28	A	17 (25)	307
SHV-33	A	2 (3)	35
SHV-36	A	1 (1.5)	253
SHV-99	A	5 (7)	321
CTX-M-15	A	35 (51)	11, 16, 37, 273, 307, 512, 2623, 3227
LEN-7	A	2 (3)	512, 3227
LEN-12	A	9 (13)	512, 3227
OKP-B3	A	2 (3)	416
VIM-1	B	9 (13)	253, 321, 416
NDM-1	B	3 (4.5)	11
OXA-1	D	40 (59)	16, 37, 273, 307, 512, 2623, 3227
OXA-9	D	33 (48)	35, 307, 512, 1519, 2623, 3227
OXA-23	D	2 (3)	512

## Data Availability

Data sharing not applicable.

## References

[1] Bush K., Bradford P.A. (2016). β-Lactams and β-lactamase inhibitors: An overview. Cold Spring Harb. Perspect. Med..

[2] King D.T., Sobhanifar S., Strynadka N., Gotte M., Berghuis A. (2014). The mechanisms of resistance to β-lactam antibiotics. Handbook of Antimicrobial Resistance.

[3] Bush K., Bradford P.A. (2020). Epidemiology of β-Lactamase-Producing Pathogens. Clin. Microbiol. Rev..

[4] Naas T., Vandel L., Sougakoff W., Livermore D.M., Nordmann P. (1994). Cloning and sequence analysis of the gene for a carbapenem-hydrolyzing class A beta-lactamase, Sme-1, from *Serratia marcescens* S6. Antimicrob. Agents Chemother..

[5] Bush K. (2018). Past and Present Perspectives on β-Lactamases. Antimicrob. Agents Chemother..

[6] European Centre for Disease Prevention and Control (2020). Antimicrobial Resistance in the EU/EEA (EARS-Net)-Annual Epidemiological Report 2019.

[7] World Health Organization (2017). Global Priority List of Antibiotic-Resistant Bacteria to Guide Research, Discovery, and Development of New Antibiotics. https://www.who.int/medicines/publications/WHO-PPL-Short_Summary_25Feb-ET_NM_WHO.pdf.

[8] David S., Reuter S., Harris S.R., Glasner C., Feltwell T., Argimon S., Abudahab K., Goater R., Giani T., Errico G. (2019). Epidemic of carbapenem-resistant *Klebsiella pneumoniae* in Europe is driven by nosocomial spread. Nat. Microbiol..

[9] Queenan A.M., Bush K. (2007). Carbapenemases: The versatile β-Lactamases. Clin. Microbiol. Rev..

[10] Madni O., Amoako D.G., Abia A.L.K., Rout J., Essack S.Y. (2021). Genomic investigation of carbapenem-resistant *Klebsiella pneumoniae* colonization in an intensive care unit in South Africa. Genes.

[11] Zhan Q., Xu Y., Wang B., Yu J., Shen X., Liu L., Cao X., Guo Y., Yu F. (2021). Distribution of fluoroquinolone resistance determinants in carbapenem-resistant *Klebsiella pneumoniae* clinical isolates associated with bloodstream infections in China. BMC Microbiol..

[12] Ahmed M.A.E.E., Yang Y., Yang Y., Yan B., Chen G., Hassan R.M., Zhong L.L., Chen Y., Roberts A.P., Wu Y. (2021). Emergence of iypervirulent carbapenem-resistant *Klebsiella pneumoniae* coharboring a *bla(NDM-1)*-carrying virulent plasmid and a *blaKPC-2)*-carrying plasmid in an Egyptian Hospital. mSphere..

[13] Barbarini D., Russello G., Brovarone F., Capatti C., Colla R., Perilli M., Moro M.L., Carretto E. (2015). Evaluation of carbapenem-resistant Enterobacteriaceae in an Italian setting: Report from the trench. Infect. Genet. Evol..

[14] Brescini L., Morroni G., Valeriani C., Castelletti S., Mingoia M., Simoni S., Masucci A., Montalti R., Vivarelli M., Giacometti A. (2019). Clinical and epidemiological characteristics of KPC-producing *Klebsiella pneumoniae* from bloodstream infections in a tertiary referral center in Italy. BMC Infect. Dis..

[15] Martin J., Phan H.T.T., Findlay J., Stoesser N., Pankhurst L., Navickaite I., De Maio N., Eyre D.W., Toogood G., Orsi N.M. (2017). Covert dissemination of carbapenemase-producing *Klebsiella pneumoniae* (KPC) in a successfully controlled outbreak: Long- and short-read whole-genome sequencing demonstrate multiple genetic modes of transmission. J. Antimicrob. Chemother..

[16] Naas T., Cuzon G., Villegas M.V., Lartigue M.F., Quinn J.P., Nordmann P. (2008). Genetic structures at the origin of acquisition of the β-lactamase *bla*KPC gene. Antimicrob. Agents. Chemother..

[17] Garcia-Fernandez S., Villa L., Carta C., Venditti C., Giordano A., Venditti M., Mancini C., Carattoli A. (2012). *Klebsiella pneumoniae* ST258 producing KPC-3 identified in Italy carries novel plasmids and OmpK36/OmpK35 porin variants. Antimicrob. Agents. Chemother..

[18] Chen L., Chavda K.D., Melano R.G., Jacobs M.R., Levi M.H., Bonomo R.A., Kreiswirth B.N. (2013). Complete sequence of a bla(KPC-2)-harboring IncFII(K1) plasmid from a Klebsiella pneumoniae sequence type 258 strain, Antimicrob. Agents. Chemother. 2013; 57, 1542–1545. Agents. Chemother..

[19] Cerdeira L.T., Cunha M.P.V., Francisco G.R., Bueno M.F.C., Araujo B.F., Ribas R.M., Gontijo-Filho P.P., Knöbl T., de Oliveira Garcia D., Lincopan N. (2017). IncX3 plasmid harboring a non-Tn*4401* genetic element (NTE_KPC_) in a hospital-associated clone of KPC-2-producing *Klebsiella pneumoniae* ST340/CG258. Diagn. Microbiol. Infect. Dis..

[20] Endimiani A., Depasquale J.M., Forero S., Perez F., Hujer A.M., Roberts-Pollack D., Fiorella P.D., Pickens N., Kitchel B., Casiano-Colón A.E. (2009). Emergence of blaKPC-containing *Klebsiella pneumoniae* in a long-term acute care hospital: A new challenge to our healthcare system. J. Antimicrob. Chemother..

[21] Ben-David D., Masarwa S., Navon-Venezia S., Mishali H., Fridental I., Rubinovitch B., Smollan G., Carmeli Y., Schwaber M.J., Israel PACF CRKP (Post-Acute-Care Facility Carbapenem-Resistant Klebsiella pneumoniae) Working Group (2011). Carbapenem-resistant *Klebsiella pneumonia* in post-acute-care facilities in Israel. Infect. Control Hosp. Epidemiol..

[22] Giani T., Antonelli A., Caltagirone M., Mauri C., Nicchi J., Arena F., Nucleo E., Bracco S., Pantosti A., AMCLI-CoSA survey participants (2017). Evolving β-lactamase epidemiology in Enterobacteriaceae from Italian nationwide surveillance, October 2013: KPC-carbapenemase spreading among outpatients. Euro. Surveill..

[23] Chen H.Y., Jean S.S., Lee Y.L., Lu M.C., Ko W.C., Liu P.Y., Hsueh P.R. (2021). Carbapenem-Resistant Enterobacterales in Long-Term Care Facilities: A Global and Narrative Review. Front. Cell Infect. Microbiol..

[24] Lapp Z., Crawford R., Miles-Jay A., Pirani A., Trick W.E., Weinstein R.A., Hayden M.K., Snitkin E.S., Lin M.Y. (2021). Regional spread of *bla*_NDM-1_-containing *Klebsiella pneumoniae* ST147 in post-acute care facilities. Clin. Infect. Dis..

[25] Ambretti S., Bassetti M., Clerici P., Petrosillo N., Tumietto F., Viale P., Rossolini G.M. (2019). Screening for carriage of carbapenem-resistant Enterobacteriaceae in settings of high endemicity: A position paper from an Italian working group on CRE infections. Antimicrob. Resist. Infect. Control.

[26] Arena F., Vannetti F., Di Pilato V., Fabbri L., Colavecchio O.L., Giani T., Marraccini C., Pupillo R., Macchi C., Converti F. (2018). Diversity of the epidemiology of carbapenemase-producing Enterobacteriaceae in long-term acute care rehabilitation settings from an area of hyperendemicity, and evaluation of an intervention bundle. J. Hosp. Infect..

[27] Bonura C., Giuffre M., Aleo A., Fasciana T., Di Bernardo F., Stampone T., Giammanco A., Palma D.M., Mammina C., MDR-GN Working Group (2015). An update of the evolving epidemic of blaKPC carrying *Klebsiella pneumoniae* in Sicily, Italy, 2014: Emergence of multiple non-ST258 clones. PLoS ONE.

[28] Arena F., Di Pilato V., Vannetti F., Fabbri L., Antonelli A., Coppi M., Pupillo R., Macchi C., Rossolini G.M. (2020). Population structure of KPC carbapenemase-producing *Klebsiella pneumoniae* in a long-term acute-care rehabilitation facility: Identification of a new lineage of clonal group 101, associated with local hyperendemicity. Microb. Genom..

[29] Zerbino D.R. (2010). Using the Velvet de novo assembler for short-read sequencing technologies. Curr. Protoc. Bioinformatics.

[30] Larsen M.V., Cosentino S., Lukjancenko O., Saputra D., Rasmussen S., Hasman H., Sicheritz-Pontén T., Aarestrup F.M., Ussery D.W., Lund O. (2014). Benchmarking of methods for genomic taxonomy. J. Clin. Microbiol..

[31] Zankari E., Hasman H., Cosentino S., Vestergaard M., Rasmussen S., Lund O., Aarestrup F.M., Larsen M.V. (2012). Identification of acquired antimicrobial resistance genes. J. Antimicrob. Chemother..

[32] Larsen M.V., Cosentino S., Rasmussen S., Friis C., Hasman H., Marvig R.L., Jelsbak L., Sicheritz-Pontén T., Ussery D.W., Aarestrup F.M. (2012). Multilocus sequence typing of total-genome-sequenced bacteria. J. Clin. Microbiol..

[33] Carattoli A., Zankari E., García-Fernández A., Voldby Larsen M., Lund O., Villa L., Møller Aarestrup F., Hasman H. (2014). In silico detection and typing of plasmids using PlasmidFinder and plasmid multilocus sequence typing. Antimicrob. Agents. Chemother..

[34] Yang Y., Yang Y., Chen G., Lin M., Chen Y., He R., Galvão K.N., El-Gawad El-Sayed Ahmed M.A., Roberts A.P., Wu Y. (2021). Molecular characterization of carbapenem-resistant and virulent plasmids in *Klebsiella pneumoniae* from patients with bloodstream infections in China. Emerg. Microbes. Infect..

[35] Magi G., Tontarelli F., Caucci S., Sante L.D., Brenciani A., Morroni G., Giovanetti E., Menzo S., Mingoia M. (2021). High prevalence of carbapenem-resistant *Klebsiella pneumoniae* ST307 recovered from fecal samples in an Italian hospital. Future Microbiol..

[36] Giani T., Pini B., Arena F., Conte V., Bracco S., Migliavacca R., Pantosti A., Pagani L., Luzzaro F., AMCLI-CRE Survey Participants (2013). Epidemic diffusion of KPC carbapenemase-producing *Klebsiella pneumoniae* in Italy: Results of the first countrywide survey, 15 May to 30 June 2011. Euro. Surveill..

[37] Conte V., Monaco M., Giani T., D’Ancona F., Moro M.L., Arena F., D’Andrea M.M., Rossolini G.M., Pantosti A., AR-ISS Study Group on Carbapenemase-Producing *K. pneumonia* (2016). Molecular epidemiology of KPC-producing Klebsiella pneumoniae from invasive infections in Italy: Increasing diversity with predominance of the ST512 clade II sublineage. J. Antimicrob. Chemother..

[38] Peirano G., Chen L., Kreiswirth B.N., Pitout J.D.D. (2020). Emerging Antimicrobial-Resistant High-Risk Klebsiella pneumoniae Clones ST307 and ST147. Antimicrob. Agents. Chemother..

[39] Baek E.H., Kim S.E., Kim S., Lee S., Cho O.H., In Hong S., Shin J.H., Hwang I. (2020). Successful control of an extended-spectrum β-lactamase-producing *Klebsiella pneumoniae* ST307 outbreak in a neonatal intensive care unit. BMC Infect. Dis..

[40] Boonstra M.B., Spijkerman D.C.M., Voor In ‘t Holt A.F., van der Laan R.J., Bode L.G.M., van Vianen W., Klaassen C.H.W., Vos M.C., Severin J.A. (2020). An outbreak of ST307 extended-spectrum beta-lactamase (ESBL)-producing Klebsiella pneumoniae in a rehabilitation center: An unusual source and route of transmission. Infect. Control. Hosp. Epidemiol..

[41] Giufre M., Accogli M., Ricchizzi E., Barbanti F., Farina C., Fazii P., Mattei R., Sarti M., Barozzi A., Buttazzi R. (2018). Multidrug-resistant infections in long-term care facilities: Extended-spectrum beta-lactamase-producing Enterobacteriaceae and hypervirulent antibiotic resistant *Clostridium difficile*. Diagn. Microbiol. Infect. Dis..

[42] Wyres K.L., Hawkey J., Hetland M.A.K., Fostervold A., Wick R.R., Judd L.M., Hamidian M., Howden B.P., Lohr I.H., Holt K.E. (2019). Emergence and rapid global dissemination of CTX-M-15-associated *Klebsiella pneumoniae* strain ST307. J. Antimicrob. Chemother..

[43] Piccirilli A., Perilli M., Piccirilli V., Segatore B., Amicosante G., Maccacaro L., Bazaj A., Naso L., Lo Cascio G., Cornaglia G. (2020). Molecular characterization of carbapenem-resistant *Klebsiella pneumoniae* ST14 and ST512 causing bloodstream infections in ICU and surgery wards of a tertiary university hospital of Verona (northern Italy): Co-production of KPC-3, OXA-48, and CTX-M-15 β-lactamases. Diagn. Microbiol. Infect. Dis..

[44] Perilli M., Bottoni C., Pontieri E., Segatore B., Celenza G., Setacci D., Bellio P., Strom R., Amicosante G. (2013). Emergence of *bla*KPC-3-Tn4401a in *Klebsiella pneumoniae* ST512 in the municipal wastewater treatment plant and in the university hospital of a town in central Italy. J. Glob. Antimicrob. Resist..

[45] Perilli M., Bottoni C., Grimaldi A., Segatore B., Celenza G., Mariani M., Bellio P., Frascaria P., Amicosante G. (2013). Carbapenem-resistant *Klebsiella pneumoniae* harbouring *bla*_KPC-3_ and *bla*_VIM-2_ from central Italy. Diagn. Microbiol. Infect. Dis..

[46] Geraci D.M., Bonura C., Giuffrè M., Saporito L., Graziano G., Aleo A., Fasciana T., Di Bernardo F., Stampone T., Palma D.M. (2015). Is the monoclonal spread of the ST258, KPC-3-producing clone being replaced in southern Italy by the dissemination of multiple clones of carbapenem-nonsusceptible, KPC-3-producing *Klebsiella pneumoniae*?. Clin. Microbiol. Infect..

[47] Garcìa-Fernandez A., Miriagou V., Papagiannitsis C.C., Giordano A., Venditti M., Mancini C., Carattoli A. (2010). An ertapenem-resistant extended-spectrum-β-lactamase-producing *Klebsiella pneumoniae* clone carries a novel OmpK36 porin variant. Antimicrob. Agents. Chemother..

[48] Orsi G.B., Bencardino A., Vena A., Carattoli A., Venditti C., Falcone M., Giordano A., Venditti M. (2013). Patient risk factors for outer membrane permeability and KPC-producing carbapenem-resistant *Klebsiella pneumoniae* isolation: Results of a double case-control study. Infection.

[49] Oliveira É.M., Beltrão E.M.B., Scavuzzi A.M.L., Barros J.F., Lopes A.C.S. (2020). High plasmid variability, and the presence of IncFIB, IncQ, IncA/C, IncHI1B, and IncL/M in clinical isolates of *Klebsiella pneumoniae* with *blaKPC* and *blaNDM* from patients at a public hospital in Brazil. Rev. Soc. Bras. Med. Trop..

[50] Villa L., García-Fernández A., Fortini D., Carattoli A. (2010). Replicon sequence typing of IncF plasmids carrying virulence and resistance determinants. J. Antimicrob. Chemother..

[51] Ramirez M.S., Bonomo R.A., Tolmasky M.E. (2020). Carbapenemases: Transforming *Acinetobacter baumannii* into a yet more dangerous menace. Biomolecules.

[52] Arabacı Ç., Dal T., Başyiğit T., Genişel N., Durmaz R. (2019). Investigation of carbapenemase and mcr-1 genes in carbapenem-resistant *Klebsiella pneumoniae* isolates. J. Infect. Dev. Ctries..

[53] Chen L., Chavda K.D., Melano R.G., Hong T., Rojtman A.D., Jacobs M.R., Bonomo R.A., Kreiswirth B.N. (2014). Molecular survey of the dissemination of two blaKPC-harboring IncFIA plasmids in New Jersey and New York hospitals. Antimicrob. Agents. Chemother..

